# Transcriptome profiling reveals significant changes in the gastric muscularis externa with obesity that partially overlap those that occur with idiopathic gastroparesis

**DOI:** 10.1186/s12920-019-0550-3

**Published:** 2019-06-20

**Authors:** B. Paul Herring, Meng Chen, Plamen Mihaylov, April M. Hoggatt, Anita Gupta, Attila Nakeeb, Jennifer N. Choi, John M. Wo

**Affiliations:** 1 Department of Cellular and Integrative Physiology, Indiana University School of Medicine, 635 Barnhill Drive, Indianapolis, IN 46202 USA; 2 Department of Oncology Research for Biologics and Immunotherapy Translation (ORBIT), MD Anderson Cancer Center, Houston, TX 77054 USA; 3 Department of Surgery, Indiana University School of Medicine, Indianapolis, IN 46202 USA; 4 Department of Medicine, Division of Gastroenterology and Hepatology, Indiana University School of Medicine, Indianapolis, IN 46202 USA

**Keywords:** Motility, Stomach, Obesity, Gastroparesis

## Abstract

**Background:**

Gastric emptying is impaired in patients with gastroparesis whereas it is either unchanged or accelerated in obese individuals. The goal of the current study was to identify changes in gene expression in the stomach muscularis that may be contributing to altered gastric motility in idiopathic gastroparesis and obesity.

**Methods:**

Quantitative real time RT-PCR and whole transcriptome sequencing were used to compare the transcriptomes of lean individuals, obese individuals and either lean or obese individuals with idiopathic gastroparesis.

**Results:**

Obesity leads to an increase in mRNAs associated with muscle contractility whereas idiopathic gastroparesis leads to a decrease in mRNAs associated with PDGF BB signaling. Both obesity and idiopathic gastroparesis were also associated with similar alterations in pathways associated with inflammation.

**Conclusions:**

Our findings show that obesity and idiopathic gastroparesis result in overlapping but distinct changes in the gastric muscularis transcriptome. Increased expression of mRNAs encoding smooth muscle contractile proteins may be contributing to the increased gastric motility observed in obese subjects, whereas decreased PDGF BB signaling may be contributing to the impaired motility seen in subjects with idiopathic gastroparesis.

**Electronic supplementary material:**

The online version of this article (10.1186/s12920-019-0550-3) contains supplementary material, which is available to authorized users.

## Background

Gastroparesis defined as delayed gastric emptying in the absence of a physical obstruction, is estimated to occur in about 1.8% of the adult population, with the majority of cases being in women [1, 2]. Patients with gastroparesis have a marked decrease in their quality of life, with symptoms including nausea, vomiting, early satiety, bloating and abdominal pain. As gastric emptying is dependent on an intricate interaction of neural cells, interstitial cells of Cajal (ICC), platelet derived growth factor α positive (PDGFRα^+^) fibroblasts and smooth muscle cells (SMCs), it is likely that defects in any of these cell types can contribute to this disease [3–7]. ICC are the pacemaker cells in the stomach that transmit slow wave depolarization to the gastric smooth muscle cells [5, 8]. The PDGFRα^+^ fibroblasts are a more recently recognized component of the electrical syncytium that controls gastric contractility. These cells transmit inhibitory neural signals to gastric smooth muscle cells [3]. Using qRT-PCR of RNA isolated from the stomach muscularis of control and gastroparesis patients we previously reported that idiopathic gastroparesis is associated with altered smooth muscle cell contractile protein expression and loss of PDGFRα^+^ cells without a change in ICC [9]. Recently global transcriptional profiling suggested that innate immune signaling may play a central role in the pathogenesis of gastroparesis [10]. These observations are consistent with rodent studies that have shown a critical role of macrophages in the pathogenesis of gastroparesis [11, 12].

In our recent studies, and in other studies published by the NIDDK Gastroparesis Clinical Research Consortium, control biopsies have been obtained from patients undergoing bariatric weight loss surgery [4, 9, 13–15]. Although these patients provide ready access to control biopsies that can be collected from the same region of the stomach, and in the same manner as the samples from gastroparesis patients, these subjects have the complication of being obese. For example, in our recent study the mean BMI of the control subjects was 47.8 ± 8.9 compared to 25.1 ± 6.9 in the gastroparetic group [9]. Although the effects of obesity on gastric emptying are somewhat controversial a large cohort study of 328 individuals demonstrated that obese subjects (BMI > 30 kg/m^2^) have increased rates of gastric emptying as compared to low BMI subjects (BMI 18-25 kg/m^2^) [16, 17]. This increased gastric emptying in obese subjects raises the possibility that some of the molecular and cellular changes reported in patients with gastroparesis and delayed gastric emptying may be due to alterations in the obese control group rather than a consequence of gastroparesis itself. This has long been recognized as a limitation of previous gastroparesis studies. To attempt to address this limitation a preliminary analysis of histological data from obese control subjects and non-obese subjects undergoing surgery for gastroesophageal junction cancer suggested that obesity did not influence the numbers of ICC observed in muscle biopsies [13]. However, there has not been a comprehensive study performed to determine if other alterations that may occur as a result of obesity may be complicating the interpretation of changes observed in non-obese subjects with gastroparesis. This was thus the goal of the current study, in which we performed global transcriptional profiling of gastric muscularis obtained from control subjects with low BMI (organ donors) and from control subjects with high BMI (undergoing bariatric surgery). These data were then compared to data obtained from subjects with idiopathic gastroparesis who had either low (< 30) BMI or high BMI (> 30) in order to identify transcriptional changes that occur as a result of idiopathic gastroparesis and to separate these changes from those that occur as a result of obesity.

## Methods

### Clinical assessment

All procedures were conducted under protocol approved by the Indiana University Institutional Review Board. Informed consents were obtained from control subjects and patients with idiopathic gastroparesis. Idiopathic gastroparesis was defined as patients with symptoms of gastroparesis ≥3 months with delayed gastric emptying by 4-h gastric scintigraphy or the presence of a gastric bezoar with undigested foods by upper endoscopy after an overnight fast. Gastric scintigraphy was performed following consumption of a low-fat egg-white meal with imaging at 0, 2 and 4 h as described previously [18, 19]. Abnormal gastric emptying was defined as 2-h retention ≥60% or 4-h retention ≥10%. Gastroparesis patients included in the study did not have diabetes as determined by normal fasting glucose or normal hemoglobin A1c (HbA1c) by blood test. We also excluded subjects with other systemic diseases such as surgery with vagal trauma, scleroderma, mixed connective tissue disorder and paraneoplastic syndrome.

### Full-thickness stomach biopsies

Biopsies from the low BMI control group (no reported diabetes or gastroparesis) were obtained from organ transplant donors at the time of organ transplant. Biopsies from the high BMI control group were obtained from non-diabetic individuals without gastroparesis symptoms that were undergoing bariatric weight loss surgery (normal HbA1c level < 1 month prior to surgery). Surgical full-thickness biopsies from the idiopathic gastroparesis patients were obtained at the time of routine pathological analysis in patients with severe symptoms or at the time of gastric stimulator placement. In each case (controls and gastroparesis patients), the biopsy was taken about 10 cm from the pylorus on the anterior aspect midway between the greater and lesser curvatures of the stomach. All biopsies were obtained using linear cutting staplers. For sleeve gastrectomy subjects the sample was obtained after the stomach was removed from the abdomen, for all other subjects the samples were obtained laparoscopically. Tissues were collected into ice cold DMEM (Dullbeco’s modified eagle media) or RNAlater (Ambion) and immediately transferred to the investigator’s laboratory. Both the submucosal and mucosal layers were carefully removed from the muscularis by dissection under a binocular microscope, the muscularis was then flash frozen in liquid nitrogen and ground to a powder under liquid nitrogen prior to storage at − 80°. A small amount of frozen muscle tissue powder (~ 150 mg) was homogenized in Trizol (Invitrogen) and a Purelink Micro RNA isolation kit was used to isolate total RNA. Contaminating DNA was removed by on-column DNase treatment. The integrity and purity of the isolated RNA was determined using an Agilent Bioanalyzer. Samples used had an RNA integrity number of > 7. Levels of ATP4A and gastrin mRNA were analyzed in all samples by qRT-PCR to screen for mucosal contamination. Only samples which had levels of expression of these markers that were less than 100-fold lower than the levels present in mucosa were utilized for further analysis.

### Library preparation and sequencing

The concentration and quality of total RNA samples was first assessed using Agilent 2100 Bioanalyzer. A RIN (RNA Integrity Number) of 7 or higher was required to pass the quality control. Then 200 ng of RNA per sample was used to prepare a dual-indexed strand-specific cDNA library using KAPA mRNA Hyperprep Kit (Roche). The resulting libraries were assessed for their quantity and size distribution using Qubit and Agilent 2100 Bioanalyzer. Two hundred pico molar pooled libraries were utilized per flowcell for clustering amplification on cBot using HiSeq 3000/4000 PE Cluster Kit and sequenced with 2.75 bp paired-end configuration on HiSeq4000 (Illumina) using HiSeq 3000/4000 PE SBS Kit. A Phred quality score (Q score) was used to measure the quality of sequencing. More than 90% of the sequencing reads reached Q30 (99.9% base call accuracy). The sequencing was performed in the Center for Medical Genomics (CMG) at Indiana University School of Medicine, a sequencing core facility of the Indiana CTSI.

### Sequence alignment and gene counts

The sequencing data were first assessed using FastQC (Babraham Bioinformatics, Cambridge, UK) for quality control. Then all sequenced libraries were mapped to the human genome (UCSC hg38) using STAR RNA-seq aligner [20] with the following parameter: “--outSAMmapqUnique 60”. The reads distribution across the genome was assessed using bamutils (from ngsutils) [21]. Uniquely mapped sequencing reads were assigned to hg38 refGene genes using featureCounts (from subread) with the following parameters: “-s 2 –p –Q 10” [22]. Quality control of sequencing and mapping results was summarized using MultiQC [23]. Genes with read count per million (CPM) > 0.5 in more than 4 of the samples were kept. The data was normalized using TMM (trimmed mean of M values) method. The median number of uniquely mapped reads obtained per sample were 28.7 million (86.96% of raw reads) with a minimum of 22.3 million and a maximum of 32 million. Differential expression analysis was performed using edgeR [24, 25]. False discovery rate (FDR) was computed from *p*-values using the Benjamini-Hochberg procedure. Complete RNA-sequencing data is available from the NCBI GEO database, accession number GSE129398.

qRT-PCR. 500 ng of RNA was used in reverse transcription reactions (High Capacity RT-cDNA Kit, Life Technologies). Real time PCR was performed using Sybr Green (Roche). Levels of mRNA expression were normalized to expression of TATA binding protein (TBP) as an internal control and are expressed relative to the mean values seen in samples from control low BMI patients. The primers used for qRT-PCR are listed in Additional file 4: Table S1

### Statistical analysis

Significant differences between groups were identified using one way Anova analysis with a Kruskal-Wallis test and a Duns Multiple Comparison post-test.

## Results

### Obese subjects have elevated ICC markers but no change in markers of PDGFRα positive fibroblasts compared to low BMI subjects

**Fig. 1:**
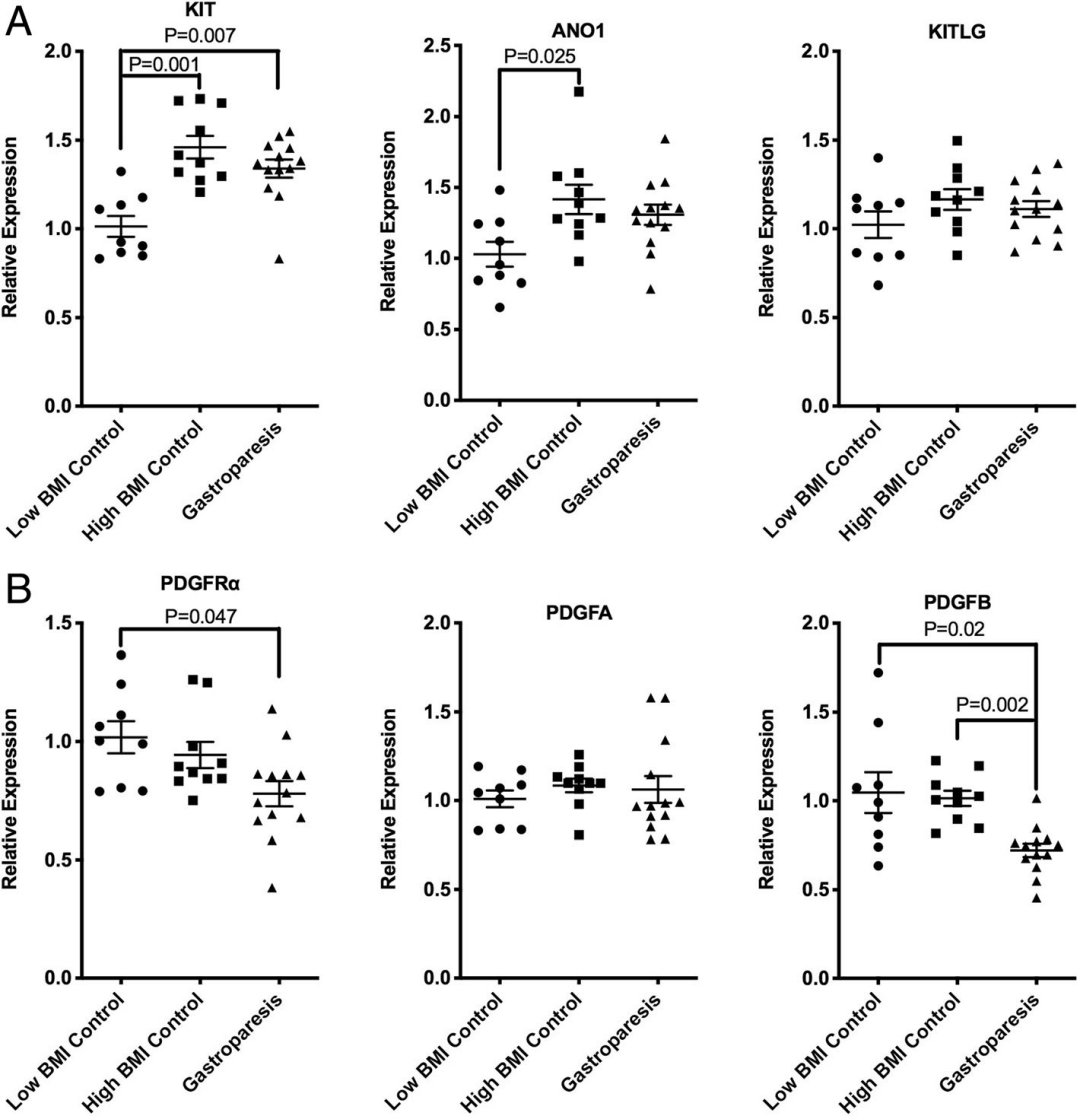
Increased KIT and ANO1 mRNA in obese subjects, decreased PDGFRα and PDGFB mRNA in subjects with gastroparesis**.** Total RNA was isolated from the muscle layer of biopsies obtained from 9 control subjects with low BMI, 10 control subjects with high BMI and 13 subjects with idiopathic gastroparesis and low BMI. qRT-PCR was used to measure the expression of each of the mRNAs indicated. Data presented were normalized to an internal control encoding TBP and are expressed relative to levels in the control low BMI subjects. Relative expression =2^-ΔΔCt^, where ΔΔCt = (Ct_High BMI or Gastroparesis_-Ct_TBP_) – (Ct_Low BMI control_-Ct_TBP_). Each point represents an individual subject. The mean ± SEM are also indicated. Significant differences between groups were identified using one-way Anova analysis with a Kruskal-Wallis test and a Duns Multiple Comparison post-test

To determine if obesity is affecting the transcriptome of the stomach muscularis externa we obtained full thickness biopsy samples from transplant donors at the time of organ harvest and reevaluated the previously reported mRNA changes described in idiopathic gastroparesis subjects [9]. Tissues were obtained from low BMI control donors (Mean BMI 24.0 ± 2.8, mean age 34.8 ± 13, 4 female, 5 male, *n* = 9), high BMI control bariatric surgery patients (Mean BMI 49.2 ± 10, mean age 43.7 ± 7.5, 8 female, 2 male, *n* = 10) and low BMI subjects with idiopathic gastroparesis (mean BMI 23.2 ± 3.8, mean age 35.5 ± 10.6, 11 female, 2 male *n* = 13). The clinical data of the subjects used are summarized in Additional file 5: Table S2 Previous immune-histological studies have suggested that ICCs are decreased in patients with idiopathic gastroparesis [4, 13]. However, we did not observe any changes in mRNA encoding the ICC markers KIT or ANO1 in our previous study, although we did observed decreased expression of PDGFRα and PDGFB mRNA and in mRNAs encoding several smooth muscle contractile proteins [9]. We thus reevaluated these findings utilizing samples from low BMI transplant donors as controls. This analysis revealed that the ICC markers KIT and ANO1 were significantly higher in the muscularis of control subjects with high BMI as compared to those with low BMI (Fig. 1a). KIT mRNA was also significantly higher in idiopathic gastroparesis subjects with low BMI, compared to the low BMI control patients. Consistent with our previous studies there was no significant difference in these mRNAs between the high BMI control subjects and the idiopathic gastroparesis patients. In contrast to KIT and ANO1 mRNA expression, PDGFRα and PDGFB mRNAs were not significantly changed in high BMI control subjects as compared to the low BMI control subjects (Fig. 1b). However, these mRNAs were significantly decreased in the low BMI gastroparesis subjects compared to the low BMI control subjects or high BMI control subjects (Fig. 1b).

### mRNAs encoding some smooth muscle contractile proteins were elevated both in obese subjects and in subjects with gastroparesis

**Fig. 2:**
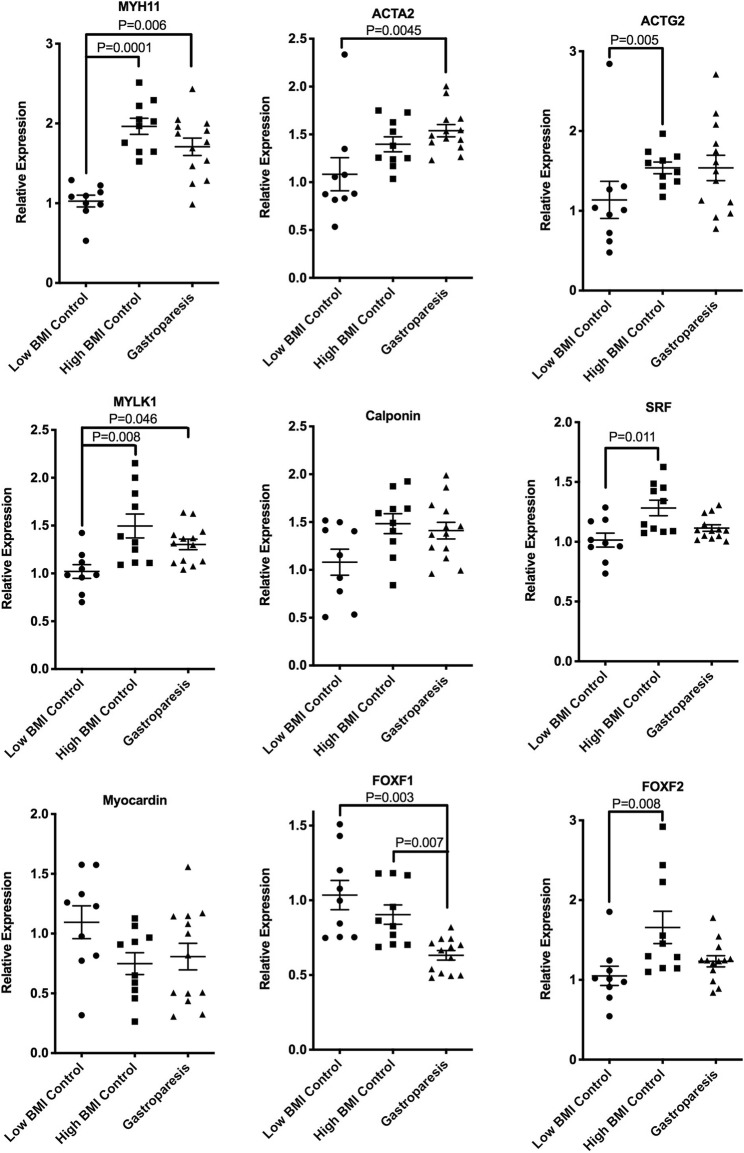
mRNAs indicative of contractile smooth muscle are elevated in the muscularis of obese subjects and in subjects with gastroparesis**.** Total RNA was isolated from the muscle layer of biopsies obtained from 9 control subjects with low BMI, 10 control subjects with high BMI and 13 subjects with idiopathic gastroparesis and low BMI. qRT-PCR was used to measure the expression of each of the indicated mRNAs. Data presented were normalized to an internal control encoding TBP and are expressed relative to levels in the control low BMI subjects. Relative expression =2^-ΔΔCt^, where ΔΔCt = (Ct_High BMI or Gastroparesis_-Ct_TBP_) – (Ct_Low BMI control_-Ct_TBP_). Each point represents an individual subject. The mean ± SEM are also indicated. Significant differences between groups were identified using one-way Anova analysis with a Kruskal-Wallis test and a Duns Multiple Comparison post-test

In contrast to our previously reported decreases in expression of mRNAs encoding some contractile proteins in idiopathic gastroparesis subjects compared to high BMI control subjects [9], when compared to low BMI transplant donor controls, idiopathic gastroparesis subjects had elevated levels of mRNAs encoding MYH11, ACTA2 and MYLK1 (Fig. 2). Interestingly MYH11, MYLK1 and ACTAG2 mRNAs were also significantly elevated in high BMI control subjects compared to low BMI control subjects, this was accompanied by a significant elevation in SRF mRNA (Fig. 2). This elevation in the high BMI control group could account for our previous finding of decreased expression of these mRNAs in idiopathic gastroparesis subjects as compared to high BMI control subjects [9].

### Obesity and gastroparesis are associated with distinct but overlapping gastric muscularis transcriptomes

**Fig. 3:**
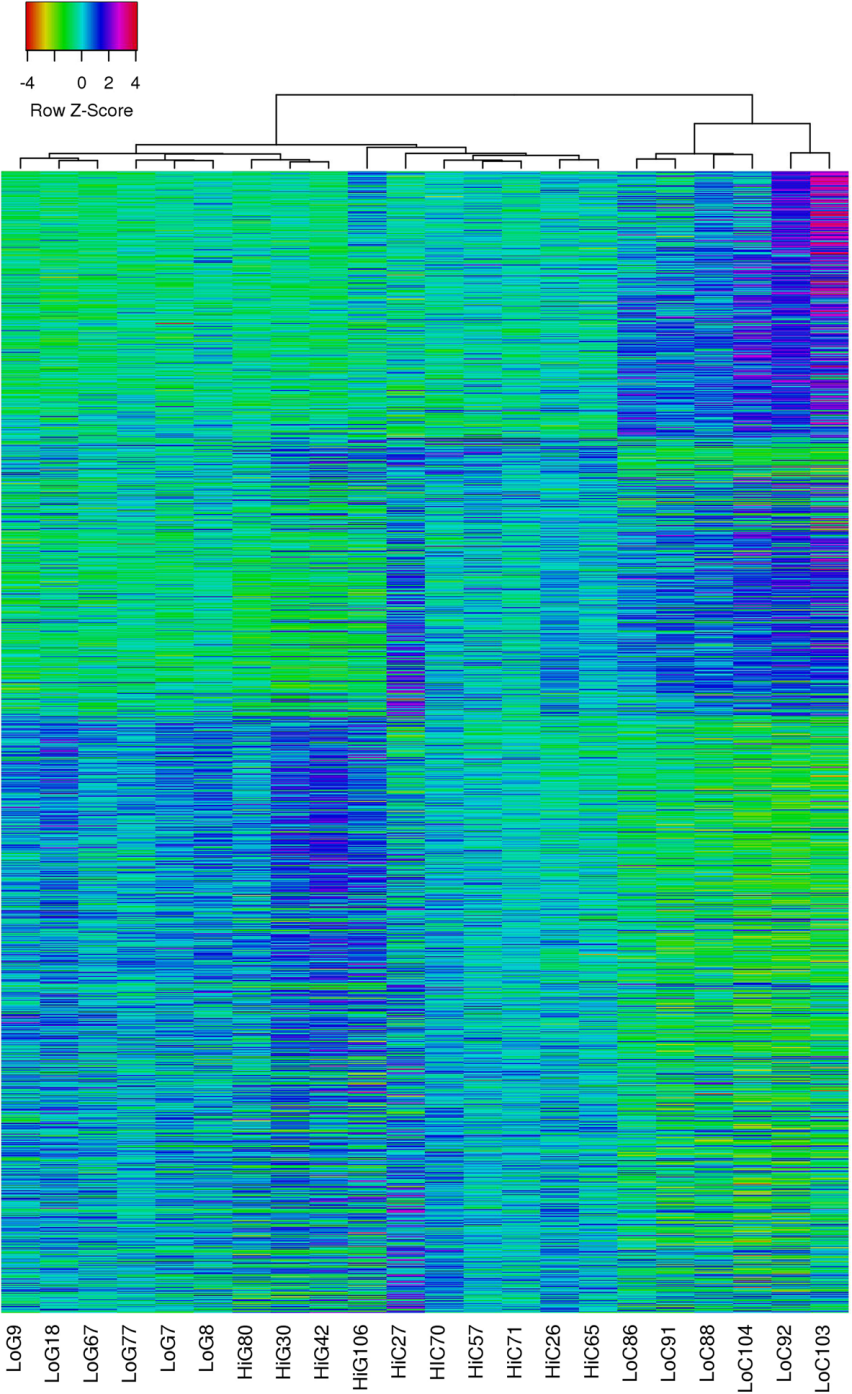
Heatmap linkage analysis illustrates distinct differences in the transcriptome in control, obese and gastroparesis subjects. To visualize the overall differences in mRNA expression between each of the groups of samples used for RNA sequencing we performed a heat map analysis on all genes that were found to be significantly different between any group of samples as determined by an ANOVA analysis with an FDR cutoff of < 0.05. Expression data were exported from edgeR software and average log2-counts-per-million were imported into Heatmapper (www.heatmapper.ca). For the heat map analysis sample grouping was assessed using a complete linkage analysis together with a Pearson distance analysis. LoC- low BMI control, HiC-high BMI control, LoG- low BMI gastroparesis, HiG-high BMI gastroparesis

Together, the data described above, suggest that obesity has a significant impact on mRNA expression in the stomach muscularis. These findings may significantly alter the interpretation of previous data that utilized tissues obtained from obese patients as controls. For example, increased expression of mRNAs in stomach muscularis of obese subjects may result in an apparent decrease in these mRNAs in gastroparesis subjects when in fact they may be unchanged or increased compared to low BMI control subjects. To evaluate this possibility more fully we performed a whole transcriptome RNA-sequencing analysis of the muscularis obtained from low BMI control donors (Mean BMI 24.8 ± 3.3, mean age 38.5 ± 15.7, 3 female and 3 male, *n* = 6), high BMI control bariatric surgery patients (Mean BMI 51.9 ± 11.5, mean age 44.8 ± 8.9, 3 female and 3 male, n = 6) and low or high BMI subjects with gastroparesis (mean BMI 24.2 ± 4.6, mean age 41 ± 7.9, all female, n = 6 and mean BMI 36.6 ± 1.8, mean age 43 ± 17.9, 3 female and 1 male, *n* = 4, respectively). To visualize the overall differences in mRNA expression between each of the groups of samples we performed a heat map analysis on all genes that were found to be significantly different between any group of samples as determined by an ANOVA analysis with an FDR cutoff of < 0.05. For the heat map analysis sample grouping was assessed using a complete linkage analysis together with a Pearson distance analysis (Heatmapper). This analysis clearly showed that the samples obtained from control patients with a low BMI clustered away from both the samples from control patients with high BMI and the samples from subjects with idiopathic gastroparesis (Fig. 3). Interestingly the high BMI control samples clustered closer to the idiopathic gastroparesis samples than the low BMI control samples. As expected the samples obtained from control subjects with high BMI were also distinct from those obtained from subjects with gastroparesis regardless of the BMI of the gastroparesis subjects. Although the samples from high and low BMI idiopathic gastroparesis subjects separated from each other they were more similar to each other than to either of the control groups (Fig. 3).

**Fig. 4:**
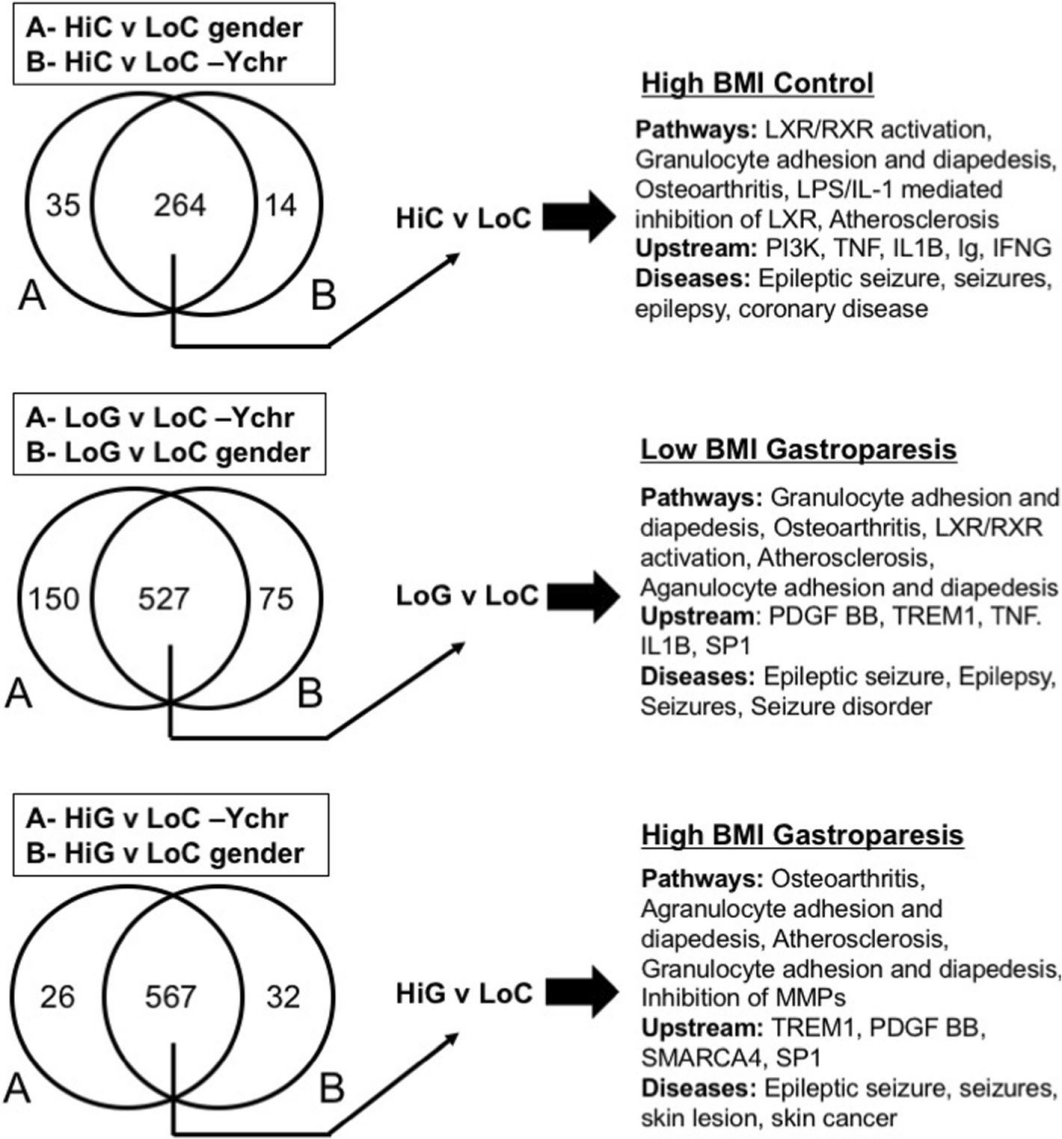
Pathway analysis of differentially expressed genes. Genes differentially expressed between either the high BMI control group (HiC) or the low (LoG) or high (HiG) BMI gastroparesis groups and the low BMI control group (LoC) were analyzed in two different ways. In the first, log2 fold changes were calculated on all samples relative to the low BMI samples, the resultant gene sets were then filtered to remove genes located on the Y chromosome. In a second approach samples were compared only to other samples obtained from subjects of the same gender (Additional file 6 : Table S3). Differentially expressed genes were then filtered to those exhibiting a logFC of − 1 or less or of + 1 or greater with a FDR of < 0.05 (i.e. genes with a 2 or greater fold change in expression). A comparison analysis was then performed to identify those genes whose change in expression was significant no matter which analysis was performed. These lists of genes were then subject to Ingenuity pathway analysis. The resultant top 5 canonical pathways, upstream regulators and diseases are shown

**Fig. 5:**
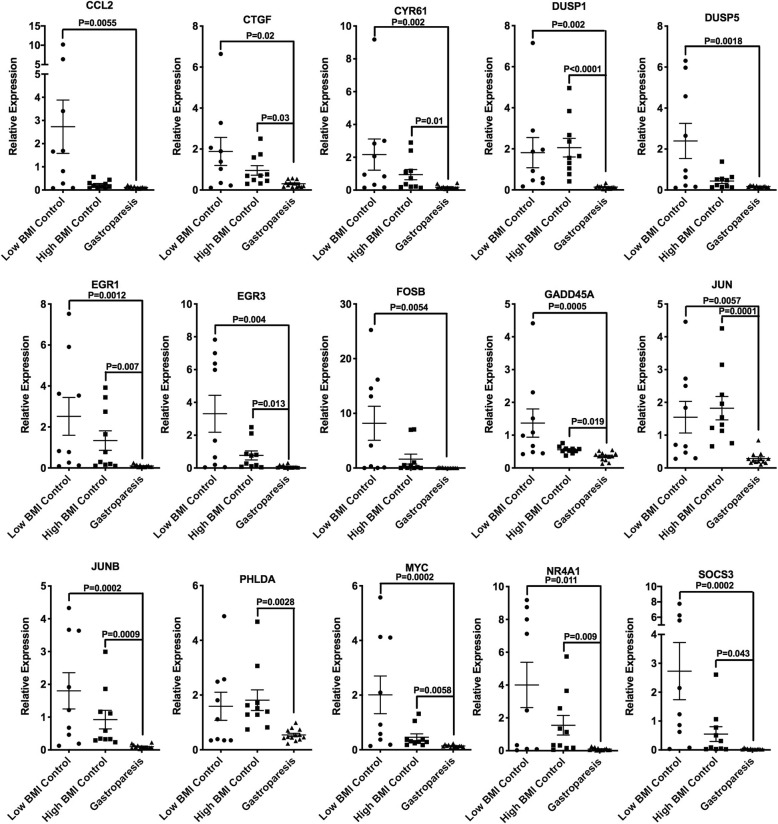
Predicted PDGF BB regulated genes are downregulated in gastroparesis subjects**.** Consistent with the qRT-PCR data shown in Fig. 1b, Ingenuity pathway analysis revealed that PDGF BB signaling may be altered in subjects with gastroparesis (Fig. 4 and Additional file 7: Table S4). Total RNA was isolated from the muscle layer of biopsies obtained from 9 control subjects with low BMI, 10 control subjects with high BMI and 13 subjects with idiopathic gastroparesis and low BMI. qRT-PCR was used to verify some of these changes in PDGF BB dependent mRNAs. Data presented were normalized to an internal control encoding TBP and are expressed relative to levels in the control low BMI subjects. Relative expression =2^-ΔΔCt^, where ΔΔCt = (Ct_High BMI or Gastroparesis_-Ct_TBP_) – (Ct_Low BMI control_-Ct_TBP_). Each point represents an individual subject. The mean ± SEM are also indicated. Significant differences between groups were identified using one-way Anova analysis with a Kruskal-Wallis test and a Duns Multiple Comparison post-test

To begin to identify which changes in mRNA expression were associated with gastroparesis as opposed to obesity we first compared differentially expressed genes in each group as shown in Fig. 4. Differentially expressed genes were analyzed in two different ways, in the first, log2 fold changes were calculated on all samples relative to the low BMI samples, the resultant gene sets were then filtered to remove genes located on the Y chromosome. In a second approach samples were compared only to other samples obtained from subjects of the same gender (Additional file 6: Table S3) Differentially expressed genes were then filtered to those exhibiting a logFC of − 1 or less or of + 1 or greater with a FDR of < 0.05 (i.e. genes with a 2 or greater-fold change in expression). Within these data, we identified those genes whose expression was altered irrespective of which method of analysis we used (Y chromosome filtered or gender based) and then compared those genes identified in each group (Fig. 4). From this analysis, we identified 264 genes that were differentially expressed in the high BMI control group compared to the low BMI control group, 527 genes that were differentially expressed in the low BMI gastroparesis group compared to the low BMI control group and 567 genes that were differentially expressed in the high BMI gastroparesis group compared to the low BMI control group (> 2 fold, FDR < 0.05, Fig. 4). Ingenuity pathway analysis of each of these groups of genes revealed a marked similarity in canonical pathways affected with, granulocyte adhesion and diapedesis, osteoarthritis and atherosclerosis signaling being within the top 5 affected pathways shared between all groups (Fig. 4). This finding is consistent with the previously reported studies which reported immune dysregulation in human gastroparesis and the general idea that obesity is associated with inflammation [10]. It is however, important to note that the about two thirds of the genes within these pathways that were significantly changed were down-regulated rather than being upregulated, in samples from obese patients and in samples from patients with gastroparesis. Despite the similarities in affected pathways in the obese and gastroparesis subjects, Ingenuity analysis identified distinct potential upstream regulators that were affected in each group. For example, PI3K was the most confidently predicted upstream regulator of genes in the high BMI control group compared to the low BMI control group. Several of the predicted PI3K target genes were further examined by qRT-PCR, of these CXCL10 was found to be specifically elevated in high BMI control subjects, whereas HMOX1 and SREBF1 were decreased both in high BMI control subjects and in low BMI subjects with idiopathic gastroparesis (Additional file 1: Figure S1). In contrast, PDGF BB and TREM1 were the most significant predicted upstream regulators of the idiopathic gastroparesis groups. As our initial qRT-PCR analysis revealed decreased PDGFRα and PDGFB in gastroparesis subjects (Fig. 1b) we further validated a number of the mRNAs predicted to be regulated by PDGF BB (Additional file 7: Table S4) This analysis confirmed the downregulation of almost all the predicted PDGF BB targets that we examined and revealed that these changes were specific to the idiopathic gastroparesis subjects as none of these target genes were significantly affected by obesity (Fig. 5).

**Fig. 6:**
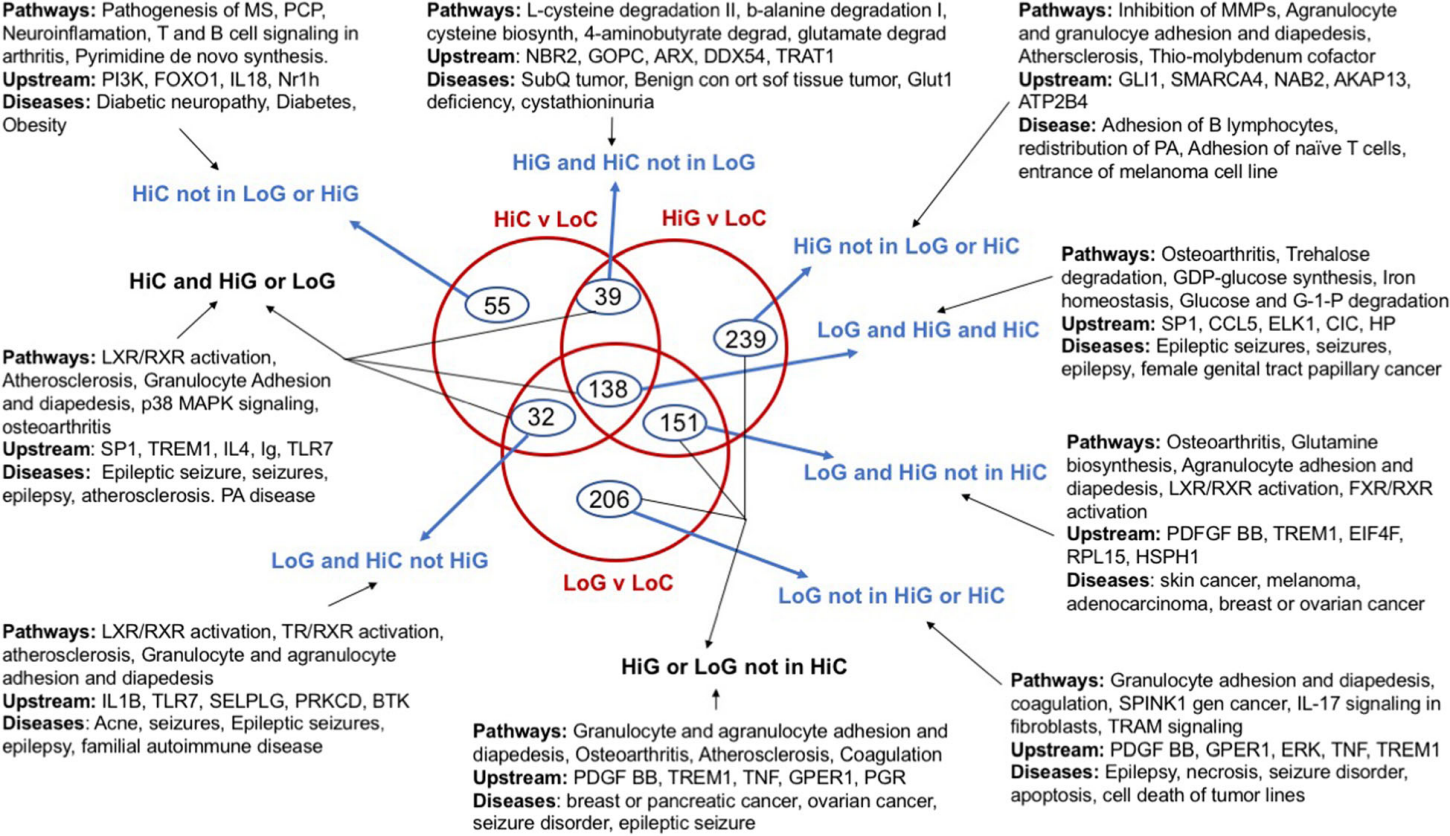
Comparison of the changes in mRNA expression in each of the subject groups**.** Comparison analysis and pathway analysis were performed using Ingenuity Pathway Analysis software. The Venn Diagram shown illustrates the numbers of mRNAs whose expression are altered 2-fold or more (FDR < 0.05) in the control subjects with high BMI (HiC) or in the gastroparesis subjects with low (LoG) or high BMI (HiG) as compared to low BMI control subjects (LoC). Ingenuity pathway analysis was performed on each of the indicted groups of genes from each segment of the Venn diagram and the top 5 canonical pathways, upstream regulators and diseases are indicated

In light of the similarities in canonical pathways affected in each of our groups of subjects we further investigated the overlap of the genes that were significantly altered in each group. The Venn diagram shown in Fig. 6 illustrates the overlaps between the genes whose expression were significantly different in samples from high BMI control subjects and low or high BMI subjects with gastroparesis as compared to the low BMI control subjects. We compiled 9 lists of differentially expressed genes representing the genes in each segment of the Venn diagram (Additional file 8: Table S5) Ingenuity pathway analysis was then performed on each of these gene lists to identify the canonical pathways, upstream activators and diseases that were enriched in each group (Fig. 6). From this analysis, we noted that the mRNAs that were differentially expressed in the high BMI control samples but not affected by gastroparesis (55 mRNAs) were significantly related to diabetic neuropathy, diabetes and obesity as might be anticipated (HiC not in LoG or HiG; Fig. 6). Of the mRNAs altered in the gastroparesis groups that were not also altered in the high BMI control group only 151 were shared between the high and low BMI gastroparesis groups, whereas 239 were unique to the high BMI gastroparesis group and 206 unique to the low BMI gastroparesis group (Fig. 6). These three groups of mRNAs combined were enriched for pathways associated with granulocyte and agranulocyte adhesion and diapedesis, osteoarthritis and atherosclerosis (HiG or LoG not in HiC; Fig. 6). This group was also enriched for the upstream activators PDGF BB and TREM1 and the diseases, breast, pancreatic or ovarian cancer and seizure disorders. These observations are consistent with the findings that many PDGF BB target genes are uniquely affect by gastroparesis but not obesity (Fig. 5).

**Fig. 7:**
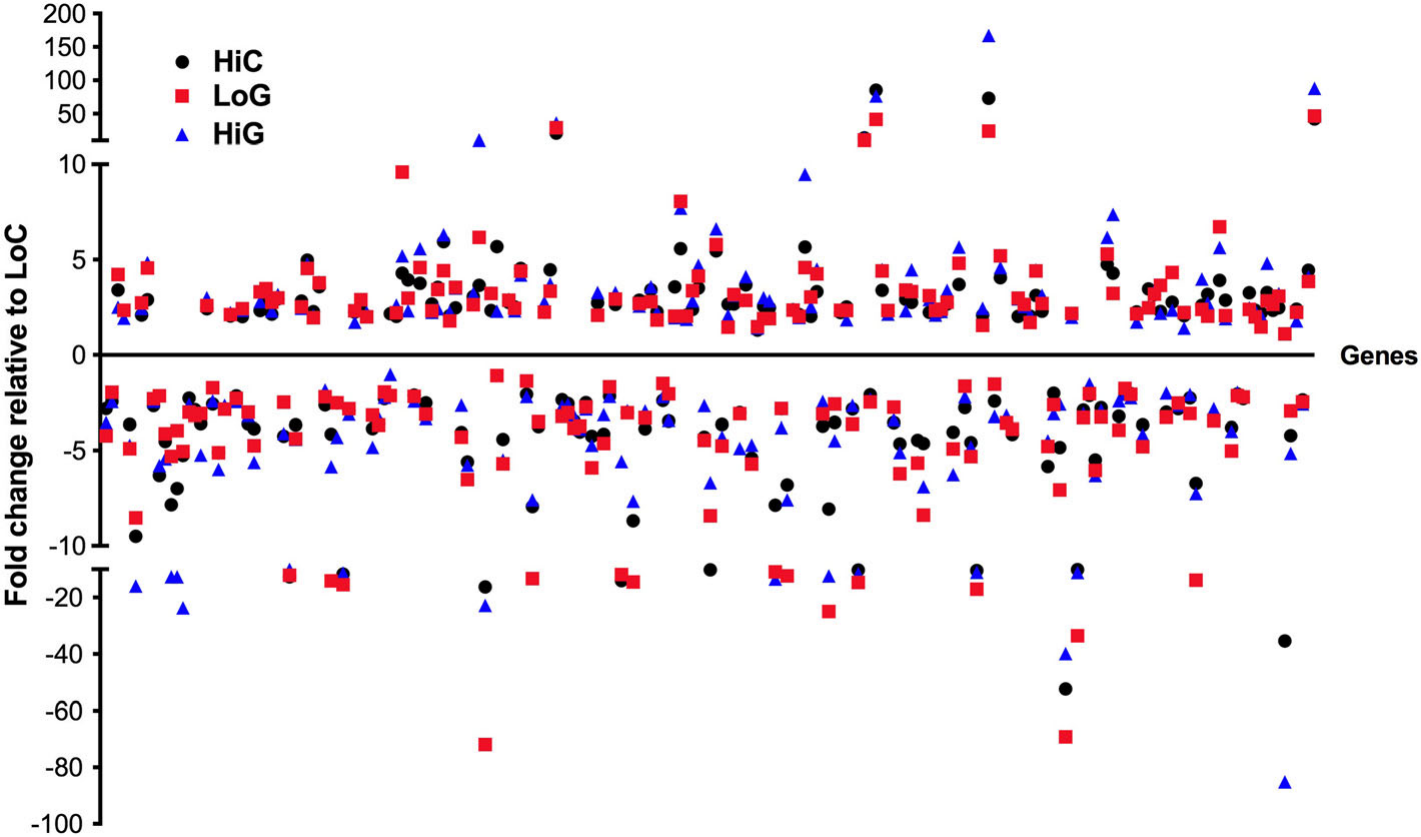
Both obesity and gastroparesis have similar effects on a core group of genes. Relative changes in expression of the common set of mRNAs whose expression was significantly altered in control subjects with high BMI (HiC) and in gastroparesis subjects with low(LoG) or high BMI (HiG) are indicated (The, LoG and HiG and HiC group of 138 genes shown in Fig. 6)

Pathway analysis of the 209 mRNAs shared between the high BMI control group and either the low or high BMI gastroparesis groups revealed an enrichment in pathways associated with LXR/RXR activation in addition to those associated with granulocyte adhesion and diapedesis, atherosclerosis and osteoarthritis (HiC and HiG or LoG; Fig. 6). Interestingly the most enriched upstream activator in this group was SP1. As obesity has been reported to be associated with an increased rate of gastric emptying as compared to the decreased gastric emptying characteristic of gastroparesis we hypothesized that if genes within this shared group are related to gastric emptying then they may be oppositely affected in the obesity and gastroparesis subjects. However, we found that almost all of genes within this shared group were similarly affected by both obesity and gastroparesis suggesting that they are not likely to be directly related to gastric emptying (Fig. 7).

### Transcriptome changes associated with gastroparesis irrespective of the BMI of control subjects

A recent study reported results of a similar transcriptome wide analysis of changes in gene expression in the stomach of gastroparesis patients [10]. There are two major differences between this published study and the current study. Firstly, we utilized muscularis which was dissected free from mucosa and submucosa from full thickness biopsies to harvest RNA for our analysis whereas in the previous study the mucosa was simply peeled from the biopsy. Secondly, we utilized low BMI transplant donors as our control group whereas the previous study utilized subjects undergoing bariatric surgery and therefore presumably with high BMIs. To better compare the findings of the two studies we reanalyzed our data without separating the low and high BMI gastroparesis groups from each other (CombG group in Additional file 2: Figure S2; Additional file 9: Table S6) We then compared the differentially expressed genes (relative to the low BMI controls) identified in our CombG group, to the differentially expressed genes in the high BMI control group and those identified in the idiopathic gastroparesis group of the previous published study (Grover; Additional file 2: Figure S2). Of the 657 differentially expressed genes in the CombG group only 157 were also identified in the previous study and 41 of these were also differentially expressed in the high BMI control group (HiC, CombG and Grover group; Additional file 2: Figure S2). Pathway analysis of the 116 mRNAs that were shared between the CombG group and the previous study but not in the high BMI control group revealed an enrichment in pathways associated with coagulation, the role of macrophages, fibroblasts and endothelial cells in rheumatoid arthritis, granulocyte adhesion and diapedesis and atherosclerosis (Grover and CombG not in HiC group; Additional file 2: Figure S2). PDGF BB was again identified as the most enriched upstream activator of this group consequently many of the most differentially expressed genes are predicted PDGF BB targets and have been verified in Fig. 5. We further validated changes in 6 additional mRNAs within this group by qRT-PCR (Additional file 3: Figure S3). Surprisingly this analysis failed to validate the most highly upregulated gene in this group, PRKG2. It might be predicted that the 360 genes whose expression was altered in the published idiopathic gastroparesis cohort but not altered in either our high BMI controls or our idiopathic gastroparesis subjects may represent genes that are changed selectively in the submucosa as this was not rigorously removed in the biopsies used in the previous study (Grover not HiC or CombG group; Additional file 2: Figure S2). Pathway analysis of these genes revealed an enrichment in genes associated with Th1/Th2 activation, phagosome formation, B cell receptor signaling and CD28 signaling (Grover not in HiC or CombG; Additional file 2: Figure S2). Predicted enriched upstream activators of this group include TNF, PDGF BB and IL2 and disease processes associated with this group include, cell movement, activation of cells, activation of blood cells, leukopoiesis and binding of blood cells (Additional file 2: Figure S2).

## Discussion

The results of our study show that obesity results in changes in mRNA expression in the gastric muscularis that reflects increased expression of markers of ICC and smooth muscle cells and a general decrease in expression of genes associated with inflammation (Figs. 1 and 2). We also observed an increase in mRNAs encoding contractile proteins in the gastric muscularis of subjects with idiopathic gastroparesis and confirmed our previously reported decreases in mRNAs associated with PDGFRα-positive fibroblasts in these subjects (Figs. 1 and 2). Altered PDGF BB signaling was further suggested from our RNA-sequencing analysis which revealed PDGF BB as a predicted upstream regulator of many of the mRNAs whose expression were specifically decreased in idiopathic gastroparesis subjects (Figs. 4 and 6, Additional file 7: Table S4).

Immuno-histological studies have suggested that numbers of ICC are decreased in patients with idiopathic and diabetic gastroparesis [13, 14, 26] However, our previous quantitative RT-PCR analysis failed to detect any changes in mRNAs characteristic of ICC (KIT, ANO1) [9]. The current study suggests that the control samples used in all the previous studies which were obtained from subjects undergoing bariatric surgery may actually have increased expression of mRNAs characteristic of ICC compared to control subjects with a low BMI (Fig. 1a). This may suggest that the reported decreases in ICC in idiopathic gastroparesis subjects may actually reflect an increase of ICC in the high BMI control subjects relative to the generally low BMI idiopathic gastroparesis subjects. However, not consistent with this interpretation is our previous and current study in which we did not detect any significant differences in expression of KIT, ANO1 or KITLG between the high BMI control subjects and the low BMI idiopathic gastroparesis subjects. Moreover, there was a significant increase in KIT mRNA expression in the idiopathic gastroparesis subjects compared to the low BMI control subjects. Together our data would suggest that as both obesity and idiopathic gastroparesis result in increased KIT mRNA expression yet these two conditions have opposing effects on gastric emptying, changes in KIT mRNA expression do not correlate with changes in gastric emptying. A limitation of our current and previous studies is that we are using changes in mRNA expression to make inferences about abundance of ICC cells, we did not directly measure ICC abundance, additionally protein levels of these ICC markers may not directly reflect mRNA levels. Conversely, the previous immune-histological studies measured numbers of ICC based on the number of KIT positive cells but did not directly measure overall KIT expression. How changes in ICC number or mRNA/protein expression affect overall ICC function and pacemaker activity will be an important question to unravel in order to fully elucidate the role of ICC in idiopathic gastroparesis.

We previously reported decreases in mRNAs encoding smooth muscle contractile proteins in the gastric muscularis of subjects with idiopathic gastroparesis as compared to high BMI control subjects [9]. However, when compared to control subjects with low BMI, mRNAs encoding contractile proteins were not decreased in low BMI idiopathic gastroparesis subjects and in fact several were significantly increased (Fig. 2). These mRNAs were also increased in the high BMI control subjects relative to the low BMI control subjects (Fig. 2). As obesity may be associated with increased gastric emptying [16, 17], the increased expression of mRNAs encoding smooth muscle contractile proteins could contribute to increased gastric motility in obese subjects. These increases in mRNAs encoding smooth muscle contractile proteins were also associated with increased expression of serum response factor (SRF) mRNA, a key transcription factor that regulates expression of many of these genes. In contrast, it is clear that increased expression of contractile proteins in idiopathic gastroparesis subjects is not likely to be resulting in impaired gastric emptying. The reason behind the increases in several of these mRNAs encoding contractile proteins in the idiopathic gastroparesis subjects is not so obvious. One possible explanation could be that this represents a compensatory mechanism that is activated in order to try to increase motility in subjects with impaired gastric emptying. This would be a very difficult mechanism to test in humans as it would be necessary to obtain biopsies from gastroparesis subjects early in the progression of their disease prior to the initiation of compensatory mechanisms, which is not clinically feasible.

Although we observed that obesity has significant effects on the mRNAs characteristic of ICC and smooth muscle cells it did not affect expression of the three mRNAs we examined that would be characteristic of PDGFRα -positive fibroblasts (Fig. 1b). Both PDGFRα and PDGFB were downregulated in idiopathic gastroparesis subjects relative to low BMI control subjects, whereas their expression was not significantly different between low and high BMI control subjects. Decreased PDGF BB signaling in idiopathic gastroparesis subjects was also suggested by upstream activator analysis of RNA-sequencing data and subsequent qRT-PCR confirmation (Figs. 4 and 6, Additional file 7: Table S4) This analysis further confirmed that these changes were specific to the idiopathic gastroparesis and were not also induced by obesity alone. Together these data provide a strong argument that decreased PDGF BB signaling may be associated with the pathogenesis of idiopathic gastroparesis. However, this must be interpreted within the limitations of our study. We have not directly measured PDGF BB signaling but rather analyzed expression of predicted target genes as a surrogate, and all these target genes are also regulated by other signaling pathways. From our studies, it is also not possible to determine if the predicted impairment in PDGF BB signaling is restricted to the PDGFRα-positive fibroblasts or if it also occurs in other cell types, as PDGF BB can also signal through PDGFRB which is expressed on other cells, including gastric smooth muscle cells [27]. Despite these limitations, given the known impact of PDGF BB on smooth muscle cell proliferation together with the importance of PDGFRα-positive fibroblasts in inhibitory neuromuscular transmission and the fact that many of the target genes we confirmed as being decreased are related to proliferation it is not unreasonable to propose that decreased PDGF BB signaling may be contributing to the pathogenesis of idiopathic gastroparesis.

Extensive data from rodent models and in vitro studies support the proposal that altered macrophage polarization plays a critical role in the pathogenesis of gastroparesis [11, 12, 28]. Recent human studies further support this model in showing that there is an increased percentage of mRNAs related to M1 macrophages in subjects with idiopathic gastroparesis [10]. The majority of the canonical pathways affected by gastroparesis in the current study were also related to inflammation. However, we noted that many of the genes in these pathways were downregulated. Closer examination of these genes revealed that they represent both proinflammatory cytokines and receptors such as IL1B, IL1R1/2, CCL2, SPP1 and ICAM as well as inhibitory molecules such as IL1RN and SOCS3. Surprisingly, similar decreases in inflammation related genes were observed in the gastric muscularis of obese subjects. This was unexpected as obesity is generally known to be an inflammatory state. Again, however, the decreases in mRNAs encoding both pro- and anti-inflammatory cytokines prevent us from determining if there is an overall increase or decrease in inflammation in the gastric muscularis. The decrease in HMOX1 which encodes heme-oxidase 1 an enzyme expressed in M2 macrophages that helps protect against oxidative stress would be consistent with previous studies suggesting an increase in the M1/M2 macrophage ratio in gastroparesis. (Additional file 1: Figure S1) [10, 28, 29] We attempted to further interrogate the inflammatory cell changes using Cibersort [30], however, none of our samples had significant *P* values in this analysis preventing us from drawing any meaningful conclusions from the data. We hypothesize that this may be due to the relatively low abundance of inflammatory cells in our samples, as a result of the removal of the mucosa and submucosa from our biopsies which would be where most of the immune cells would be expected to be located. Consistent with this idea is our observation that the group of mRNAs identified in the previous study by Grover et al. that were not significantly altered in our idiopathic gastroparesis subjects, and therefore may represent mRNAs from the submucosa, were enriched for immune signaling pathways (Th1/Th2 activation, phagosome formation, B cell receptor signaling and CD28 signaling, Additional file 2: Figure S2) [10].

Our overall approach of identifying mRNAs whose expression are altered in idiopathic gastroparesis irrespective of the BMI of the gastroparesis subjects should identify changes that are most commonly associated with the disease and thus most likely to be related to its underlying pathophysiology. Use of Ingenuity pathway analysis to identify pathways associated with the changes in mRNA expression may, however, introduce some bias in the identified pathways as a result of an overabundance of molecules linked to cancer and inflammation in the Ingenuity database. The approach we used also has the limitation in that it does not permit the identification of molecules or pathways that are crucial to the pathophysiology of gastroparesis in only individual subjects or subgroups of subjects. In addition, the relatively small sample size used in both the qRT-PCR analysis and the RNA-sequencing analysis limits the detection of relatively small gene expression changes that may also be further masked by unknown comorbidities in the patients. A much more extensive analysis, using a far greater number of gastroparesis subjects will be required to determine if there are subgroups of subjects that have shared transcriptional changes, which could account for the unique clinical manifestations of their disease.

## Conclusions

Results from our study suggest that it may be better to utilize BMI matched control subjects when investigating pathological changes that occur during idiopathic gastroparesis. Data suggest that obesity alone may be inducing changes in the stomach muscularis that increase motility and affect inflammation. Analysis suggest that altered PDGF BB signaling may be acting together with dysfunctional inflammatory pathways in the pathogenesis of idiopathic gastroparesis.

## Additional files

Additional file 1: **Figure S1.** qRT-PCR verification of PI3K/SP1 regulated genes. (JPG 1660 kb)

Additional file 2: **Figure S2.** Comparison of the changes in mRNA expression observed in the current study to those previously reported by Grover et al. (JPG 490 kb)

Additional file 3: **Figure S3.** qRT-PCR verification of additional genes with altered expression. (JPG 2440 kb)

Additional file 4: **Table S1.** Primers used for qRT-PCR assays. (DOCX 169 kb)

Additional file 5: **Table S2.** Clinical summary of subjects studied. The table lists the subjects used in the study, their BMI, age, sex and 4-h gastric retention. ND-not determined. (XLSX 37 kb)

Additional file 6: **Table S3.** RNA- sequencing data. The table shows the list of samples used for RNA-sequencing, mapping statistics for each sample and differential gene expression analysis either with Y-chromosome encoded genes filtered out (DE analysis –Ychr del) or when samples were only compared between subjects of the same sex (DE analysis –gender). (XLSX 30240 kb)

Additional file 7: **Table S4.** Summary of changes in expression of predicted PDGF BB target genes obtained from RNA-sequencing data. PDGF BB target genes that were altered in the Low BMI gastroparesis subjects relative to the low BMI control subjects with a FDR < 0.05 are indicated, together with the predicted PDGF BB target genes identified in the previously published Idiopathic Gastroparesis cohort [10]. (XLS 28 kb)

Additional file 8: **Table S5.** List of genes in each segment of the Venn Diagram shown in Fig. 6. (XLS 63 kb)

Additional file 9: **Table S6.** Reanalysis of RNA sequencing data following combining low and high BMI gastroparesis subjects into one group. (XLSX 13418 kb)

## Data Availability

The datasets generated during the current study are included in this published article (and its supplemental information) and are available in the NIH GEO repository under accession number GSE129398.

## Abbreviations

BMI: Body mass index
ICC: Interstitial cells of Cajal
PDGFRα+ fibroblasts: PDGFRα positive fibroblasts
SMC: Smooth muscle cell
