# Supplementary figures and images for: Transcriptome profiling reveals significant changes in the gastric muscularis externa with obesity that partially overlap those that occur with idiopathic gastroparesis
Source: BMC Med Genomics. 2019 Jun 20;12:89. doi: 10.1186/s12920-019-0550-3 (PMC6587273; doi:10.1186/s12920-019-0550-3)

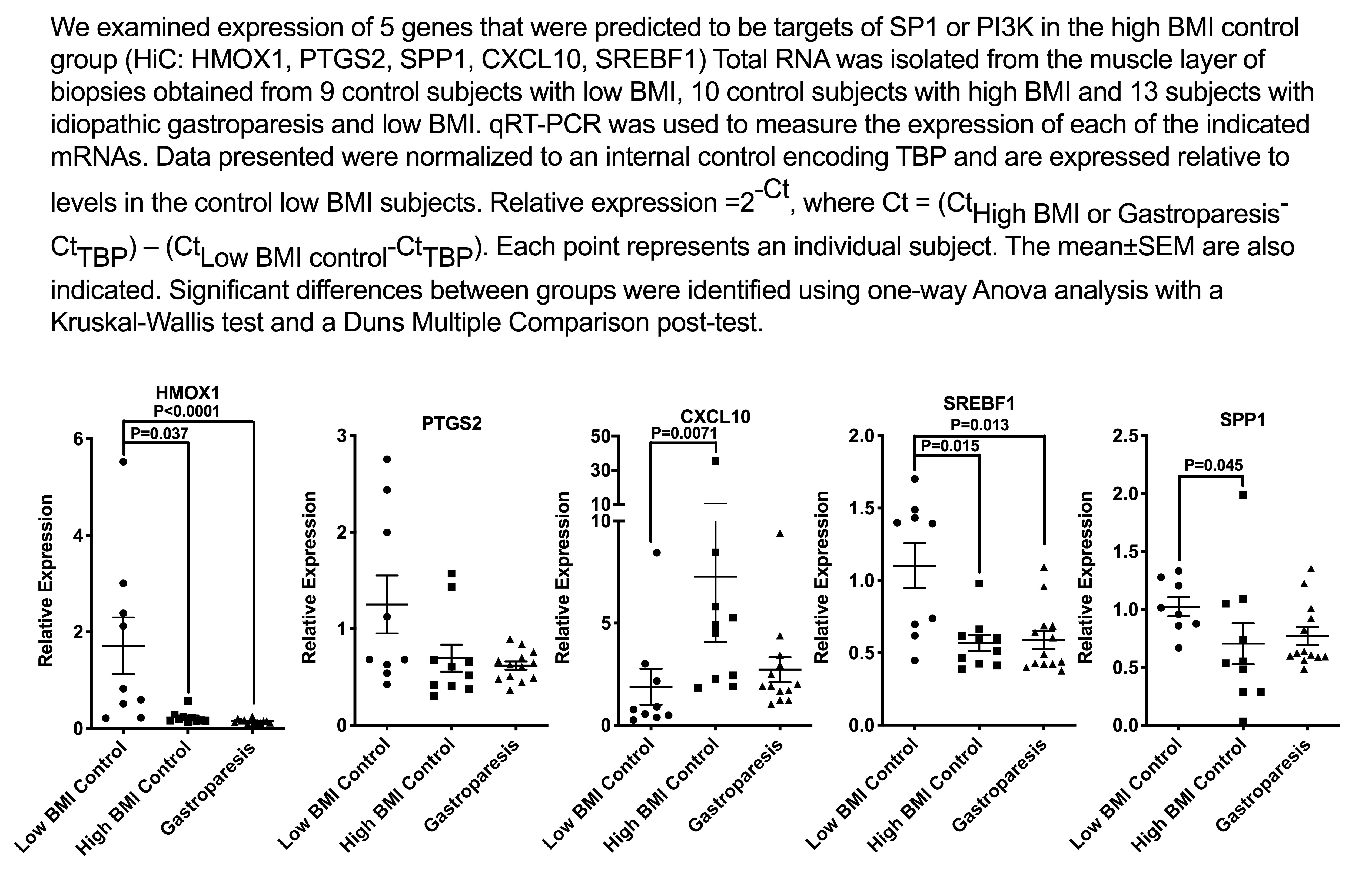

Supplement: Supplementary file 1 — Figure S1. qRT-PCR verification of PI3K/SP1 regulated genes. (JPG 1660 kb) [file 12920_2019_550_MOESM1_ESM.jpg]

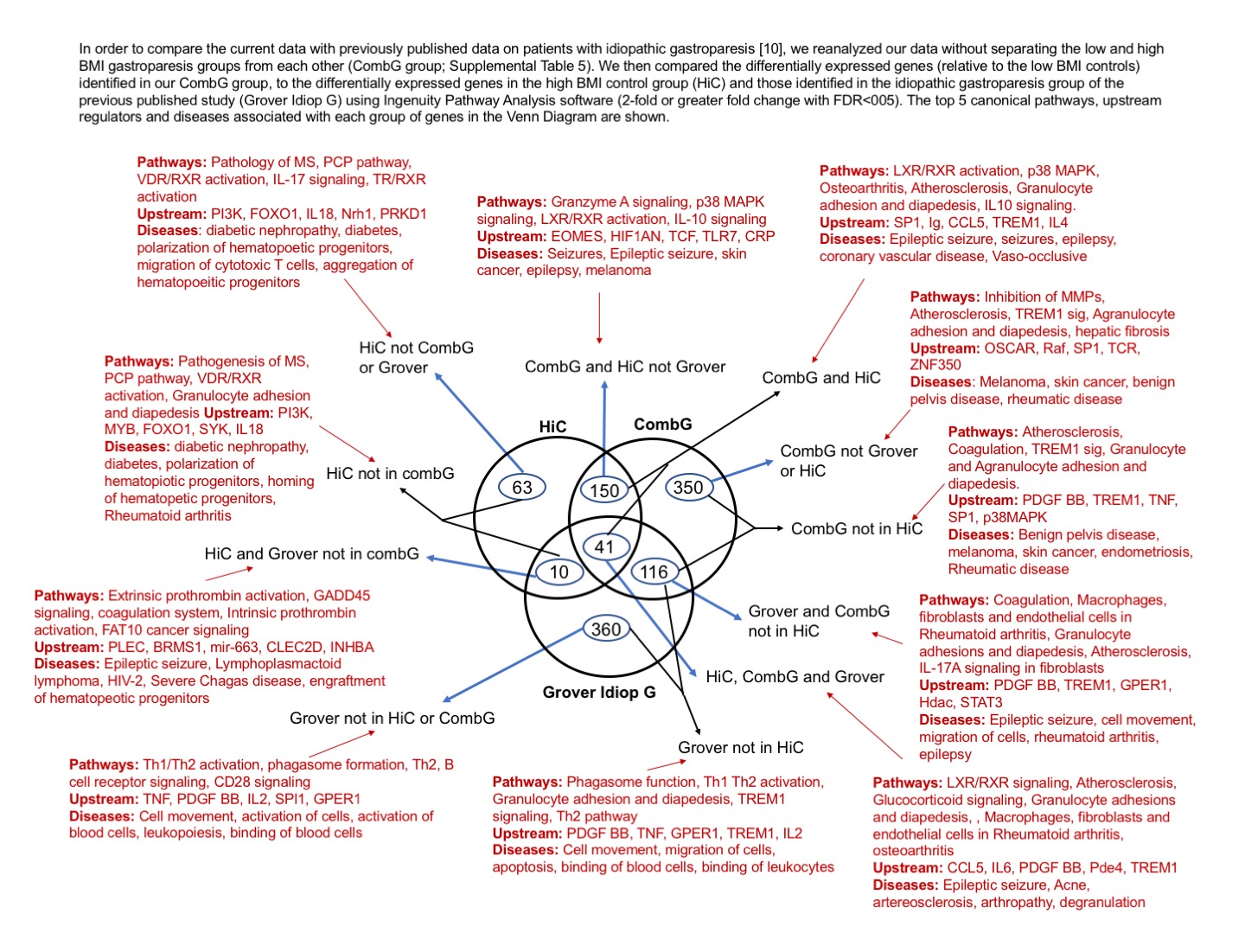

Supplement: Supplementary file 2 — Figure S2. Comparison of the changes in mRNA expression observed in the current study to those previously reported by Grover et al. (JPG 490 kb) [file 12920_2019_550_MOESM2_ESM.jpg]

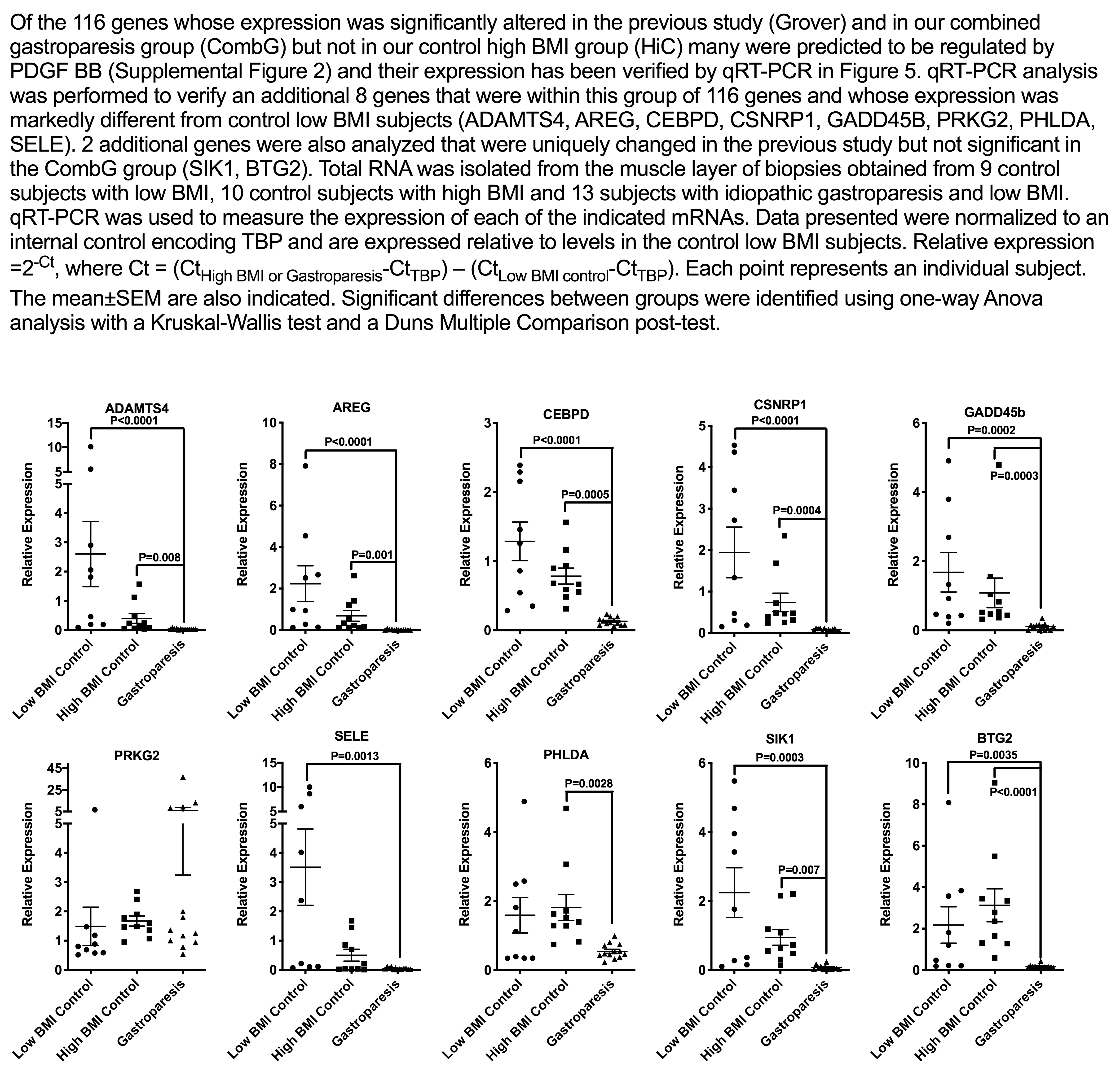

Supplement: Supplementary file 3 — Figure S3. qRT-PCR verification of additional genes with altered expression. (JPG 2440 kb) [file 12920_2019_550_MOESM3_ESM.jpg]
