# Supplementary material for: Transcriptome profiling reveals significant changes in the gastric muscularis externa with obesity that partially overlap those that occur with idiopathic gastroparesis
Source: BMC Med Genomics. 2019 Jun 20;12:89. doi: 10.1186/s12920-019-0550-3 (PMC6587273; doi:10.1186/s12920-019-0550-3)
Supplement: Supplementary file 4 — Table S1. Primers used for qRT-PCR assays. (DOCX 169 kb) [file 12920_2019_550_MOESM4_ESM.docx]

**Supplemental Table 1.** Primers used for qPCR.

| Gene | Sense | Antisense |
| --- | --- | --- |
| ACTA2 | CTGTTCCAGCCATCCTTCAT | TCATGATGCTGTTGTAGGTGGT |
| ACTG2 | CCAGCTCACTCAGCCACACACACC | CCACCATCACACCCTGGTGGCGAG |
| ADAMTS4 | ccaggcactgggctactact | aacagggggtcccatcta |
| ANO1 | GGTTCCCAGCCTAGGTCA | AGGGGGAGGAGTTCATGG |
| AREG | tgatcctcacagctgttgct | tccattctcttgtcgaagtttct |
| ATP4A | CCATCGTGGTGGCCTATG | CTGTCAGGGACAGGCAGACT |
| BTG2 | gcgagcagaggcttaaggt | gggaaaccagtggtgtttgta |
| CCL2 | AGTCTCTGCCGCCCTTCT | GTGACTGGGGCATTGATT |
| CCN1 | CCACACGACATTTTTGAGGC | ATTTCCGCTCCTGCTTCTCTGC |
| CEBPD | ggacataggagcgcaaagaa | gcttctctcgcagtttagtgg |
| CSNRP1 | cctgcctgaccgtgactt | agcccgcttcaggataga |
| CTGF | ctcctgcaggctagagaagc | gatgcactttttgcccttctt |
| CYR61 | aagaaacccggatttgtgag | gctgcatttcttgcccttt |
| DUSP1 | ggacaaccacaaggcagac | tccagcattcttgatggagtc |
| DUSP5 | acaaatggatccctgtggaa | cctcccttttccctgacac |
| EGR1 | TCCATATTAGGGCTTCCTGC | TTTCAAGGTCTGGAACAGC |
| EGR3 | caatctgtaccccgaggaga | ccgatgtccattacattctctg |
| FOSB | GAC TCC TTC GGC AGT CCA C | GTT CC GGC ATG TCG TAG |
| FOXF1 | CAGCCTCTCCACGCACTC | CCTTTCGGTCACACATGCT |
| FOXF2 | AGAGCTACTTGCACCAGAACG | CCATTGAAGTTGAGGACGAAA |
| GAST | AGAGACCTGAGAGGCACCAG | ACATACACACATAGTCGCTGCAT |
| GADD45A | agagcagaagaccgaaagga | tgactcagggctttgctga |
| GADD45B | cggccaacttgatgaatgt | gatttgcagggcgatgtc |
| HMOX1 | GGCAGAGGGTGATAGAAGAGG | AGCTCCTGCAACTCCTCAAA |
| IL1B | TACCTGTCCTGCGTGTTGAA | TCTTTGGGTAATTTTTGGGATCT |
| JUN | ccaaaggatagtgcgatgttt | ctgtccctctccactgcaac |
| JUNB | atacacagctacgggatacgg | gctcggtttcaggagtttgt |
| KIT | ATGGCATGCTCCAATGTGT | GGCAGTACAGAAGCAGAGCA |
| KITLG | GCGCTGCCTTTCCTTATG | CCTTCAGTTTTGACGAGAGGA |
| MYH11 | GCAACGCCAAAACAGTGAAGAAC | TGTGGAATGTCCTCTCGTCTCTG |
| MYLK1 | CTCCTGCTACTTTCCTTTTTCCCTACACTG | CATCAGCACCAACTCCTCCACCACAG |
| MYOCD | TATGGACTCAGCCTACGCTGCGCTG | GTATTGCTCAGTGGCGTTGAAGAAGAG |
| MYC | tgctccatgaggagacacc | cctcatcttcttgttcctcca |
| NR4A1 | acagcttgcttgtcgatgtc | ggttctgcagctcctccac |
| PDK4 | ttgggaaaagaagaccttacca | ggcggtcaataattctcagg |
| PDGFA | CAGTCAGATCCACAGCATCC | CAGGCTGGTGTCCAAAGAAT |
| PDGFB | CTGGCATGCAAGTGTGAGAC | CGAATGGTCACCCGAGTTT |
| PDGFRA | AGGTGGTTGACCTTCAATGG | TTTGATTTCTTCCAGCATTGTG |
| PHLDA | cctccaactctgcctgaaag | aaatgtgctcgtcccacttc |
| PRKG2 | caaggagaaccaggaaacca | tttctccccttggaacacct |
| PTGS2 | cttcacgcatcagtttttcaag | tcaccgtaaatatgatttaagtccac |
| SELE | caagttcgcctgtcctgaa | gggaatgttggactcagtgg |
| SIK1 | cgtcggacacctcactgac | tcccagaaacccttcctc |
| SOCS3 | agacttcgattcgggacca | aacttgctgtgggtgaccat |
| SPP1 | gagggcttggttgtcagc | caattctcatggtagtgagttttcc |
| SREBF1 | cgctcctccatcaatgaca | tgcgcaagacagcagattta |
| SRF | AGCACAGACCTCACGCAGA | GTTGTGGGCACGGATGAC |
| TBP | TTGGGTTTTCCAGCTAAGTTCT | CCAGGAAATAACTCTGGCTCA |
